# 3-[4-(Dimethyl­amino)phen­yl]-1-(2-pyrrol­yl)prop-2-en-1-one

**DOI:** 10.1107/S1600536808014700

**Published:** 2008-05-21

**Authors:** Si-Ping Tang, Dai-Zhi Kuang, Yong-Lan Feng, Wei Li, Zhi-Min Chen

**Affiliations:** a Department of Chemistry and Materials Science, Hengyang Normal University, Hengyang, Hunan 421008, People’s Republic of China

## Abstract

The molecule of the title compound, C_15_H_16_N_2_O, is non-planar with a dihedral angle of 16.0 (1)° between the pyrrole and benzene rings. The ketone double-bond displays an *s–cis* conformation with an O=C—C=C torsion angle of 7.9 (3) and an intramolecular C—H⋯O hydrogen bond. In the crystal structure, adjacent mol­ecules are paired through N—H⋯O hydrogen bonds into centrosymmetric dimers.

## Related literature

For the pharmaceutical and biological activities of chalcones, see: Lin *et al.* (2002 ▶); Lunardi *et al.* (2003 ▶); Modzelewska *et al.* (2006 ▶); Opletalova (2000 ▶); Opletalova & Sedivy (1999 ▶); Sogawa *et al.* (1994 ▶). For the use of chalcones as photonic materials, see: Balaji *et al.* (2003 ▶); Indira *et al.* (2002 ▶).
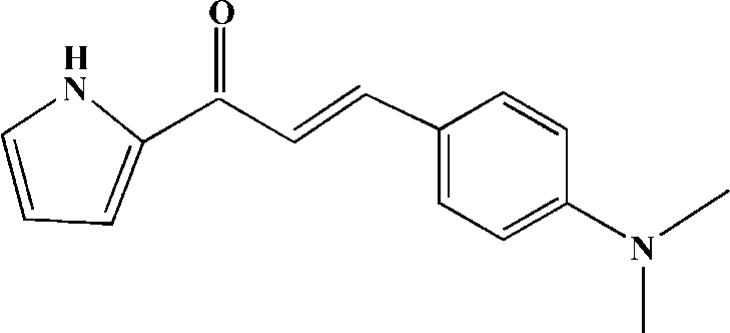


## Experimental

### 

#### Crystal data

C_15_H_16_N_2_O
*M* *_r_* = 240.30
Monoclinic, 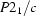

*a* = 11.0864 (16) Å
*b* = 12.0412 (17) Å
*c* = 10.6169 (16) Å
β = 112.294 (2)°
*V* = 1311.3 (3) Å^3^
*Z* = 4
Mo *K*α radiation
μ = 0.08 mm^−1^
*T* = 293 (2) K
0.20 × 0.18 × 0.17 mm

#### Data collection

Bruker APEX area-detector diffractometer
Absorption correction: multi-scan (*SADABS*; Sheldrick, 1996 ▶) *T*_min_ = 0.985, *T*_max_ = 0.991
6889 measured reflections
2568 independent reflections
1654 reflections with *I* > 2σ(*I*)
*R*_int_ = 0.040

#### Refinement

*R*[*F* ^2^ > 2σ(*F* ^2^)] = 0.065
*wR*(*F* ^2^) = 0.159
*S* = 1.09
2568 reflections
165 parameters
H-atom parameters constrained
Δρ_max_ = 0.13 e Å^−3^
Δρ_min_ = −0.15 e Å^−3^

### 

Data collection: *SMART* (Bruker, 2002 ▶); cell refinement: *SAINT* (Bruker, 2002 ▶); data reduction: *SAINT*; program(s) used to solve structure: *SHELXS97* (Sheldrick, 2008 ▶); program(s) used to refine structure: *SHELXL97* (Sheldrick, 2008 ▶); molecular graphics: *SHELXTL* (Sheldrick, 2008 ▶); software used to prepare material for publication: *SHELXTL*.

## Supplementary Material

Crystal structure: contains datablocks I, global. DOI: 10.1107/S1600536808014700/ez2122sup1.cif

Structure factors: contains datablocks I. DOI: 10.1107/S1600536808014700/ez2122Isup2.hkl

Additional supplementary materials:  crystallographic information; 3D view; checkCIF report

## Figures and Tables

**Table 1:** Hydrogen-bond geometry (Å, °)

*D*—H⋯*A*	*D*—H	H⋯*A*	*D*⋯*A*	*D*—H⋯*A*
N1—H1*A*⋯O1^i^	0.86	2.01	2.832 (2)	161
C7—H7⋯O1	0.93	2.44	2.797 (3)	103

Symmetry code: (i) 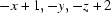.

**Fig. 1.**
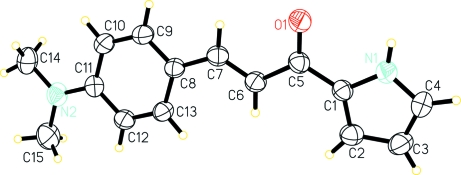
The molecular structure of (I) showing the atom numbering scheme, with displacement ellipsoids drawn at the 30% probability level, and H atoms as spheres of arbitrary radius.

**Fig. 2.**
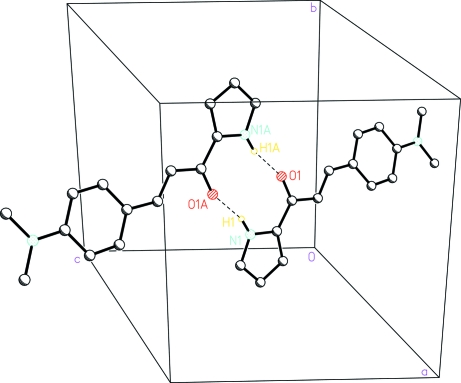
Partial packing diagram of the title structure showing the N—H···O hydrogen bonding interactions as dashed lines. H atoms not involved in hydrogen bonding have been omitted for clarity.

## supplementary crystallographic information

### Comment

Chalcones and their analogues are of considerable interest because they possess broad pharmaceutical (Sogawa *et al.*, 1994) and biological activities (Opletalova & Sedivy, 1999), such as anticancer (Modzelewska *et al.*, 2006), antitubercular (Lin *et al.*, 2002), trypanocidal (Lunardi *et al.*, 2003), antifungal and antibacterial properties (Opletalova, 2000). Moreover, some substituted chalcones have also been studied as negative photoresist materials (Balaji *et al.*, 2003) and non-linear optical materials (Indira *et al.*, 2002). We report here a new chalcone compound, (I), Fig. 1.

The title compound reveals an *s-cis* conformation for the O1–C5–C6–C7 [torsion angle 7.9 (3)°] ketone motif. Differently to most substituted chalcones, compound (I) is nonplanar with a dihedral angle between the pyrrole ring and benzene ring of 16.0 (1)°. In the crystal packing, the –NH groups are involved as donors to form centrosymmetric dimers through N—H···O hydrogen bonding interactions as shown in Fig. 2.

### Experimental

To a solution of 2-acetylpyrrole (1.09 g, 10.0 mmol) and 4-dimethylaminobenzaldehyde (1.49 g, 10.0 mmol) in 15 ml e thanol was added a solution of sodium hydroxide (0.40 g, 10.0 mmol) in 5 ml water at room temperature. After stirring 10 h, the solution was filtered. The resulting orange precipitate was washed with water and iced ethanol, and further recrystallized from acetone to afford orange block crystals of the title compound. Yield: 0.92 g (38.3%).

### Refinement

All H-atoms were positioned geometrically and refined using a riding model with d(C—H) = 0.93 Å, *U*_iso_=1.2*U*_eq_ (C) for aromatic and ethylene; 0.96 Å, *U*_iso_= 1.2*U*_eq_ (C) for CH_3_ atoms, and d(N—H) = 0.86 Å, *U*_iso_=1.2*U*_eq_ (N) for pyrrole nitrogen atom.

### Crystal data

**Table:**

C_15_H_16_N_2_O	*F*_000_ = 512
*M**_r_* = 240.30	*D*_x_ = 1.217 Mg m^−^^3^
Monoclinic, *P*2_1_/*c*	Mo *K*α radiation λ = 0.71073 Å
Hall symbol: -P 2ybc	Cell parameters from 1115 reflections
*a* = 11.0864 (16) Å	θ = 2.6–23.4º
*b* = 12.0412 (17) Å	µ = 0.08 mm^−^^1^
*c* = 10.6169 (16) Å	*T* = 293 (2) K
β = 112.294 (2)º	Block, orange
*V* = 1311.3 (3) Å^3^	0.20 × 0.18 × 0.17 mm
*Z* = 4	

### Data collection

**Table:**

Bruker APEX area-detector diffractometer	2568 independent reflections
Radiation source: fine-focus sealed tube	1654 reflections with *I* > 2σ(*I*)
Monochromator: graphite	*R*_int_ = 0.040
*T* = 293(2) K	θ_max_ = 26.0º
φ and ω scans	θ_min_ = 2.6º
Absorption correction: Multi-scan(SADABS; Sheldrick, 1996)	*h* = −13→13
*T*_min_ = 0.985, *T*_max_ = 0.991	*k* = −14→9
6889 measured reflections	*l* = −10→13

### Refinement

**Table:**

Refinement on *F*^2^	Secondary atom site location: difference Fourier map
Least-squares matrix: full	Hydrogen site location: inferred from neighbouring sites
*R*[*F*^2^ > 2σ(*F*^2^)] = 0.065	H-atom parameters constrained
*wR*(*F*^2^) = 0.159	*w* = 1/[σ^2^(*F*_o_^2^) + (0.0637*P*)^2^ + 0.0839*P*] where *P* = (*F*_o_^2^ + 2*F*_c_^2^)/3
*S* = 1.09	(Δ/σ)_max_ < 0.001
2568 reflections	Δρ_max_ = 0.13 e Å^−^^3^
165 parameters	Δρ_min_ = −0.15 e Å^−^^3^
Primary atom site location: structure-invariant direct methods	Extinction correction: none

### Special details

**Table:**

Geometry. All e.s.d.'s (except the e.s.d. in the dihedral angle between two l.s. planes) are estimated using the full covariance matrix. The cell e.s.d.'s are taken into account individually in the estimation of e.s.d.'s in distances, angles and torsion angles; correlations between e.s.d.'s in cell parameters are only used when they are defined by crystal symmetry. An approximate (isotropic) treatment of cell e.s.d.'s is used for estimating e.s.d.'s involving l.s. planes.
Refinement. Refinement of *F*^2^ against ALL reflections. The weighted *R*-factor *wR* and goodness of fit *S* are based on *F*^2^, conventional *R*-factors *R* are based on *F*, with *F* set to zero for negative *F*^2^. The threshold expression of *F*^2^ > σ(*F*^2^) is used only for calculating *R*-factors(gt) *etc*. and is not relevant to the choice of reflections for refinement. *R*-factors based on *F*^2^ are statistically about twice as large as those based on *F*, and *R*- factors based on ALL data will be even larger.

### Fractional atomic coordinates and isotropic or equivalent isotropic displacement parameters (Å^2^)

**Table:**

	*x*	*y*	*z*	*U*_iso_*/*U*_eq_	
O1	0.39484 (17)	0.01511 (13)	0.82369 (16)	0.0848 (6)	
N1	0.53628 (17)	0.17431 (15)	1.00559 (18)	0.0683 (6)	
H1A	0.5418	0.1100	1.0427	0.082*	
N2	−0.1228 (2)	0.10473 (18)	0.0104 (2)	0.0821 (6)	
C1	0.4663 (2)	0.19776 (18)	0.8722 (2)	0.0583 (6)	
C2	0.4841 (2)	0.30924 (19)	0.8553 (3)	0.0726 (7)	
H2	0.4480	0.3488	0.7744	0.087*	
C3	0.5650 (2)	0.3521 (2)	0.9796 (3)	0.0831 (8)	
H3	0.5933	0.4252	0.9977	0.100*	
C4	0.5953 (3)	0.2667 (2)	1.0701 (3)	0.0823 (8)	
H4	0.6483	0.2716	1.1619	0.099*	
C5	0.3924 (2)	0.11169 (19)	0.7811 (2)	0.0621 (6)	
C6	0.3125 (2)	0.14088 (19)	0.6406 (2)	0.0628 (6)	
H6	0.3205	0.2111	0.6082	0.075*	
C7	0.2285 (2)	0.06891 (18)	0.5581 (2)	0.0642 (6)	
H7	0.2257	0.0000	0.5965	0.077*	
C8	0.1409 (2)	0.08166 (17)	0.4178 (2)	0.0581 (6)	
C9	0.0498 (3)	−0.00039 (19)	0.3551 (3)	0.0776 (7)	
H9	0.0479	−0.0632	0.4052	0.093*	
C10	−0.0371 (2)	0.0067 (2)	0.2234 (2)	0.0768 (7)	
H10	−0.0960	−0.0509	0.1868	0.092*	
C11	−0.0389 (2)	0.09835 (19)	0.1430 (2)	0.0623 (6)	
C12	0.0526 (2)	0.18180 (18)	0.2049 (2)	0.0666 (6)	
H12	0.0547	0.2448	0.1550	0.080*	
C13	0.1389 (2)	0.17297 (17)	0.3365 (2)	0.0635 (6)	
H13	0.1984	0.2300	0.3733	0.076*	
C14	−0.2165 (3)	0.0175 (2)	−0.0521 (3)	0.0945 (9)	
H14A	−0.1716	−0.0517	−0.0454	0.142*	
H14B	−0.2642	0.0350	−0.1462	0.142*	
H14C	−0.2759	0.0115	−0.0061	0.142*	
C15	−0.1296 (3)	0.2014 (3)	−0.0725 (3)	0.1092 (10)	
H15A	−0.1863	0.2556	−0.0575	0.164*	
H15B	−0.1630	0.1804	−0.1667	0.164*	
H15C	−0.0440	0.2325	−0.0482	0.164*	

### Atomic displacement parameters (Å^2^)

**Table:**

	*U*^11^	*U*^22^	*U*^33^	*U*^12^	*U*^13^	*U*^23^
O1	0.1096 (14)	0.0608 (11)	0.0631 (11)	−0.0019 (9)	0.0093 (9)	0.0103 (8)
N1	0.0763 (13)	0.0619 (12)	0.0593 (12)	−0.0020 (10)	0.0172 (10)	0.0020 (9)
N2	0.0801 (14)	0.0857 (15)	0.0640 (14)	0.0035 (11)	0.0088 (11)	0.0010 (11)
C1	0.0588 (12)	0.0576 (14)	0.0562 (13)	0.0046 (10)	0.0192 (11)	0.0052 (11)
C2	0.0758 (15)	0.0639 (16)	0.0775 (18)	0.0004 (12)	0.0285 (14)	0.0081 (12)
C3	0.0896 (18)	0.0669 (16)	0.093 (2)	−0.0156 (14)	0.0349 (16)	−0.0078 (16)
C4	0.0858 (18)	0.0832 (19)	0.0690 (17)	−0.0157 (15)	0.0194 (14)	−0.0152 (15)
C5	0.0649 (14)	0.0598 (14)	0.0585 (14)	0.0063 (11)	0.0199 (11)	0.0057 (11)
C6	0.0656 (13)	0.0544 (13)	0.0620 (14)	0.0034 (11)	0.0170 (12)	0.0068 (11)
C7	0.0714 (14)	0.0535 (13)	0.0654 (15)	0.0062 (11)	0.0233 (13)	0.0068 (11)
C8	0.0620 (13)	0.0502 (13)	0.0600 (14)	0.0041 (10)	0.0207 (11)	0.0024 (10)
C9	0.0934 (18)	0.0617 (15)	0.0680 (17)	−0.0140 (13)	0.0195 (14)	0.0066 (12)
C10	0.0816 (17)	0.0723 (17)	0.0676 (17)	−0.0216 (13)	0.0182 (14)	−0.0045 (13)
C11	0.0607 (13)	0.0655 (15)	0.0574 (14)	0.0096 (11)	0.0188 (11)	−0.0016 (11)
C12	0.0732 (15)	0.0580 (14)	0.0633 (15)	0.0044 (12)	0.0199 (13)	0.0104 (11)
C13	0.0623 (13)	0.0550 (14)	0.0662 (15)	−0.0023 (10)	0.0164 (12)	0.0014 (11)
C14	0.0762 (17)	0.115 (2)	0.0791 (19)	−0.0055 (16)	0.0143 (15)	−0.0205 (16)
C15	0.116 (2)	0.114 (2)	0.0713 (19)	0.0082 (19)	0.0052 (17)	0.0173 (17)

### Geometric parameters (Å, °)

**Table:**

O1—C5	1.244 (2)	C7—H7	0.9300
N1—C4	1.339 (3)	C8—C9	1.388 (3)
N1—C1	1.361 (3)	C8—C13	1.392 (3)
N1—H1A	0.8600	C9—C10	1.367 (3)
N2—C11	1.364 (3)	C9—H9	0.9300
N2—C15	1.443 (3)	C10—C11	1.391 (3)
N2—C14	1.449 (3)	C10—H10	0.9300
C1—C2	1.379 (3)	C11—C12	1.402 (3)
C1—C5	1.442 (3)	C12—C13	1.366 (3)
C2—C3	1.383 (3)	C12—H12	0.9300
C2—H2	0.9300	C13—H13	0.9300
C3—C4	1.360 (3)	C14—H14A	0.9600
C3—H3	0.9300	C14—H14B	0.9600
C4—H4	0.9300	C14—H14C	0.9600
C5—C6	1.460 (3)	C15—H15A	0.9600
C6—C7	1.329 (3)	C15—H15B	0.9600
C6—H6	0.9300	C15—H15C	0.9600
C7—C8	1.446 (3)		
			
C4—N1—C1	109.6 (2)	C13—C8—C7	124.7 (2)
C4—N1—H1A	125.2	C10—C9—C8	123.2 (2)
C1—N1—H1A	125.2	C10—C9—H9	118.4
C11—N2—C15	122.1 (2)	C8—C9—H9	118.4
C11—N2—C14	121.4 (2)	C9—C10—C11	121.1 (2)
C15—N2—C14	116.3 (2)	C9—C10—H10	119.4
N1—C1—C2	106.5 (2)	C11—C10—H10	119.4
N1—C1—C5	120.2 (2)	N2—C11—C10	121.6 (2)
C2—C1—C5	133.3 (2)	N2—C11—C12	122.2 (2)
C1—C2—C3	108.2 (2)	C10—C11—C12	116.3 (2)
C1—C2—H2	125.9	C13—C12—C11	121.7 (2)
C3—C2—H2	125.9	C13—C12—H12	119.2
C4—C3—C2	106.8 (2)	C11—C12—H12	119.2
C4—C3—H3	126.6	C12—C13—C8	122.4 (2)
C2—C3—H3	126.6	C12—C13—H13	118.8
N1—C4—C3	109.0 (2)	C8—C13—H13	118.8
N1—C4—H4	125.5	N2—C14—H14A	109.5
C3—C4—H4	125.5	N2—C14—H14B	109.5
O1—C5—C1	119.9 (2)	H14A—C14—H14B	109.5
O1—C5—C6	121.2 (2)	N2—C14—H14C	109.5
C1—C5—C6	118.9 (2)	H14A—C14—H14C	109.5
C7—C6—C5	121.2 (2)	H14B—C14—H14C	109.5
C7—C6—H6	119.4	N2—C15—H15A	109.5
C5—C6—H6	119.4	N2—C15—H15B	109.5
C6—C7—C8	129.7 (2)	H15A—C15—H15B	109.5
C6—C7—H7	115.2	N2—C15—H15C	109.5
C8—C7—H7	115.2	H15A—C15—H15C	109.5
C9—C8—C13	115.3 (2)	H15B—C15—H15C	109.5
C9—C8—C7	120.0 (2)		
			
C4—N1—C1—C2	0.2 (3)	C6—C7—C8—C13	7.1 (4)
C4—N1—C1—C5	−179.3 (2)	C13—C8—C9—C10	−0.4 (4)
N1—C1—C2—C3	−0.1 (3)	C7—C8—C9—C10	179.2 (2)
C5—C1—C2—C3	179.4 (2)	C8—C9—C10—C11	0.1 (4)
C1—C2—C3—C4	0.0 (3)	C15—N2—C11—C10	177.3 (3)
C1—N1—C4—C3	−0.2 (3)	C14—N2—C11—C10	1.7 (3)
C2—C3—C4—N1	0.1 (3)	C15—N2—C11—C12	−4.6 (4)
N1—C1—C5—O1	−1.6 (3)	C14—N2—C11—C12	179.8 (2)
C2—C1—C5—O1	179.0 (2)	C9—C10—C11—N2	178.2 (2)
N1—C1—C5—C6	176.55 (19)	C9—C10—C11—C12	0.0 (4)
C2—C1—C5—C6	−2.9 (4)	N2—C11—C12—C13	−178.0 (2)
O1—C5—C6—C7	7.9 (3)	C10—C11—C12—C13	0.2 (3)
C1—C5—C6—C7	−170.3 (2)	C11—C12—C13—C8	−0.5 (4)
C5—C6—C7—C8	179.5 (2)	C9—C8—C13—C12	0.6 (3)
C6—C7—C8—C9	−172.4 (2)	C7—C8—C13—C12	−179.0 (2)

### Hydrogen-bond geometry (Å, °)

**Table:**

*D*—H···*A*	*D*—H	H···*A*	*D*···*A*	*D*—H···*A*
N1—H1A···O1^i^	0.86	2.01	2.832 (2)	161
C7—H7···O1	0.93	2.44	2.797 (3)	103

Symmetry codes: (i) −*x*+1, −*y*, −*z*+2.
